# Conjunctival Pigmentation Following Pars Plana Vitrectomy (PPV) for Rhegmatogenous Retinal Detachment: Risk Factors and Outcomes

**DOI:** 10.7759/cureus.36987

**Published:** 2023-03-31

**Authors:** Athanassios Dokos, Asterios Diafas, Argyrios Tzamalis, Rumana Hussain, Heinrich Heimann, Ioannis Tsinopoulos, Evangelia Tsironi, Sofia Androudi

**Affiliations:** 1 Ophthalmology, University of Thessaly, Larissa, GRC; 2 Ophthalmology, Papageorgiou General Hospital/Aristotle University of Thessaloniki, Thessaloniki, GRC; 3 Second Department of Ophthalmology, Papageorgiou General Hospital/Aristotle University of Thessaloniki, Thessaloniki, GRC; 4 Ophthalmology, Royal Liverpool University Hospital, Liverpool, GBR; 5 Second Department of Ophthalmology, School of Medicine, Faculty of Health Sciences, Aristotle University of Thessaloniki, Thessaloniki, GRC

**Keywords:** pigmented conjunctival lesion, pars plana vitrectomy (ppv), sutureless pars plana vitrectomy, retinal detachment surgery, vitreo-retinal surgeon

## Abstract

Purpose: To investigate the incidence and the risk factors for conjunctival pigmentation at the sclerotomy sites following valved and non-valved cannula pars plana vitrectomy (PPV) performed by different surgical techniques.

Methods: This is a prospective observational study which included 70 eyes of 70 patients who underwent PPV for rhegmatogenous retinal detachment with follow-up visits at 1, 3, 6, 12, and 24 months. Twenty-eight eyes were operated using 25G non-valved cannulas (Group A), 22 eyes using 25G non-valved cannulas (Group B), and 20 eyes using 25G valved cannulas (Group C). The evaluated clinical parameters include the surgical technique, the patients' age, the number of retinal tears, the tamponade agent, the presence of residual sub-retinal fluid (SRF), and the duration of postoperative posturing.

Results: Group A was associated with significant conjunctival pigmentation at up to 6 months after PPV. Sulfur hexafluoride (SF6) gas tamponade was associated with less conjunctival pigmentation at 3 months follow-up visit [odds ratio, OR 0.09 (95% confidence interval, CI 0.01; 0.67)], whereas the presence of residual SRF was a significant risk factor for postoperative pigmentation at 1-year follow-up visit [OR 5.89 (95% CI 1.84; 23.12)]. The area of measured pigmentation was also positively correlated to the number of retinal tears at all follow-up visits over 2 years. Six patients presented with conjunctival pigmentation at 2 years follow-up visit.

Conclusion: New vitrectomy techniques with valved cannulas prevent the postoperative appearance of conjunctival pigmentation. The number of retinal tears, the presence of SRF, and the use of long-standing tamponade agents were the most significant predisposing factors. The post-vitrectomy conjunctival pigmentation gradually reduces over time.

## Introduction

The evolution of vitrectomy techniques and the availability of finer surgical instruments have contributed to the establishment of small-gauge transconjunctival sutureless approaches. These advanced surgical techniques have gained popularity and have been adopted by vitreoretinal surgeons due to decreased ocular inflammation, reduced surgery time, rapid visual restoration, and enhanced vitreoretinal diseases prognosis [1]. The conventional 20G trans-scleral approach with partial conjunctival dissection which was initially introduced by O'Malley and Heintz in 1975, has been gradually replaced by the micro-incision vitrectomy surgery (MIVS) [2]. The era of MIVS started with the introduction of transconjunctival vitrectomy using 25G set of tools by Fujji et al. in 2002. Three years later Eckardt et al. presented the more flexible 23G approach making the access of the peripheral retina easier and offering improved intraoperative stability, whereas Oshima et al. developed an even smaller 27G sutureless vitrectomy system in 2012 [1, 3-6].

Initially the most common MIVS platform used was the Accurus Vitrectomy System (Alcon Surgical, Fort Worth, TX, USA) with 20G sutured sclerotomies. The transition to the next generation Constellation Vision System (Alcon Surgical, Fort Worth, TX, USA) with 23G and 25G non-valved trocars initially without sclerotomy suturing, raised many questions regarding the patient's safety and postoperative complications [7-10]. These initial concerns about the higher complication rates were not confirmed by clinical studies and reviews that showed not only a shorter operative time but also an improved safety profile and efficiency of MIVS [11-13].

The role of new smaller cannulas and their self-sealing characteristics on postoperative hypotony have also been investigated [7, 10]. Sclerotomy leakage has been reported as a reasonable explanation of conjunctival pigmentation following PPV [14-16]. Many patients who undergo either 23G or 25G sutureless transconjunctival vitrectomy have presented this postoperative clinical finding [14-15]. However, conjunctival pigmentation has also been hypothesized as a reaction to the sutures used to seal the scleral incisions following 20G conventional PPV [17]. Light and electron microscopic examination have demonstrated the presence of melanin granules in the substantia propria of the conjunctiva and in macrophages, excluding the conjunctival nevus and melanoma diagnosis [14-16].

To the best of our knowledge this is the first report in the literature that compares postoperative conjunctival pigmentation following different vitrectomy platforms and investigates possible etiological clinical factors. In the present study we estimated the presence and surface area of conjunctival pigmentation in patients with retinal detachment who were operated with different vitrectomy systems via a valved and non-valved approach. The aim of this study was also to analyze the risk factors and to determine which patients' characteristics, surgical and postoperative details predispose to conjunctival pigmentation at the sclerotomy sites.

## Materials and methods

Patients and study design

This is a prospective study including patients operated with PPV for primary retinal detachment and developed conjunctival pigmentation postoperatively. The study was conducted in accordance with the tenets of the Declaration of Helsinki and the protocol was approved by the local Biomedical Ethics Committee. A written informed consent was obtained from all patients after detailed explanation of the procedure.

Patients 18 years or older, undergoing PPV for primary rhegmatogenous retinal detachment were included in the study. All patients had to be Caucasian and willing to be followed up for at least 24 months. Patients with a history of previous surgery for retinal detachment or PPV for other indications, history of intravitreal injections or conjunctival pigmented lesions were excluded from the study. A thorough ophthalmic and systematic medical history as well as their systemic and topical medication were documented. Each patient underwent a comprehensive ophthalmic examination, including best-corrected visual acuity (BCVA), refraction, anterior segment slit-lamp examination, intraocular pressure (IOP) measurement using applanation tonometry and fundoscopy before being operated.

The patients were divided into three groups according to the surgical approach and the platform used for PPV; Alcon Accurus 25G Vitrectomy System with non-valved trocars (Group A), Alcon Constellation 25G Vitrectomy System with non-valved trocars (Group B), and Alcon Constellation 25G Vitrectomy System with valved trocars (Group C) (Alcon Surgical, Fort Worth, TX, USA). Vitrectomies were conducted with the use of non-valved cannulas in the first two groups, whereas all cases in the third group were operated with valved cannulas.

 The evaluated data and the parameters (variables) were: patient's age, method-platform of treatment, total number of retinal tears, presence of residual subretinal fluid at the end of the operation, tamponade agent used (C3F8/SF6/silicone oil), and period of postoperative posturing (face down position). All patients had anterior segment photography (Topcon SL-D7, Topcon, Tokyo, Japan) and the area of conjunctival pigmentation was measured by Image-J software (U.S. National Institutes of Health, Bethesda, MD, USA) at day 1 and at months 1, 3, 6, 12 postoperatively. The pigmented area was also evaluated when the 2-year follow-up visit was available. When more than one sclerotomy sites presented pigmentation, the sum of the pigmented areas was used for the analysis.

Surgical technique

All patients were operated by the same surgeon (S.A.). The conjunctiva was initially displaced with forceps to ensure the misalignment of scleral and conjunctival entry sites and the blade/trocar created an oblique one-step incision at the pars plana 3.5 or 4.0 mm from the limbus depending on the lens status in the supero-nasal, supero-temporal, and infero-temporal quadrants. The central vitreous gel was initially removed and the presence of posterior vitreous detachment (PVD) was checked. In cases that it was not present, PVD was surgically induced with the aid of triamcinolone. Complete core and peripheral vitrectomy were performed, whereas the vitreous base was trimmed with the help of scleral indentation. All tractions at the retinal tears and lattice degenerations were also released. Sub-retinal fluid (SRF) was drained through the main retinal break until the retina was flattened. Perfluorocarbon liquid (Bausch & Lomb Okta-line, Rochester, NY, USA) was also injected in the cases that had small, peripheral breaks. Once fluid-air exchange was performed, retinopexy treatment (cryotherapy or cryotherapy/endolaser) was applied around the retinal tears. The SF6 gas, C3F8 gas, or silicone oil were then used as intraocular tamponade agents depending on surgical indications. The removal of each cannula was followed by gentle compression over the sclerotomy wound with the cotton bud. If a sclerotomy was leaking, the entry site was sutured with Vicryl 8-0 (Ethicon, Cornelia, GA, USA), whereas 0.5 mL subconjuctival cefuroxime (1 mg/0.1 mL) and 0.5 mL dexamethasone sodium phosphate 0.4% were injected at the end of the surgery. Topical chloramphenicol 0.5% and dexamethasone sodium phosphate 0.1% eye drops were prescribed 4 times per day for 2 and 4 weeks, respectively.

Statistical analysis

Descriptive statistical analysis was carried out for the patient's characteristics and the PPV-related clinical factors. In our study a two-part model was performed by the RStudio programming environment (Core Team R, 2019, R Foundation for Statistical Computing, Vienna, Austria). Multivariate analysis was performed so as the confounding effect of multiple factors will be excluded. Two outcome variables at each time interval were used (i) zero or positive photo measurement and (ii) positive photo measurement. All potential predictors were introduced as discrete variables, whereas the number of retinal tears as a continuous variable in the multivariate analysis. The correlation between the clinical factors and the presence of postoperative conjunctival pigmentation (positive photo measurement) was analyzed by logistic regression analysis predicting the probability of occurrence of a positive value, whereas the surface area of pigmentation was evaluated by linear regression analysis estimating the level of positive values.

For the logistic regression part, the reported estimate and 95% confidence intervals (CIs) for each predictor was the odds ratio (OR), while for the linear regression part was the coefficient in the linear equation (y = aX + b, where X is a vector of covariates and “a” the coefficient), given that all the other predictors included in the model are held constant. All candidate variables were initially screened with a significance level of 0.25. Variables with p < 0.25 were then processed by the two-part model where p values <0.05 were considered as statistically significant.

## Results

A total of 86 eyes from 86 patients who underwent pars plana vitrectomy (PPV) for rhegmatogenous retinal detachment between November 2017 and October 2019 were initially enrolled in this study. Sixteen patients/eyes were excluded from the analysis. Two patients presented with pre-existing benign conjunctival epithelial melanosis, two with conjunctival nevi, three with previous ocular surgery, and four patients did not attend regular follow-up appointments and were thus excluded. Five patients were not included in the final statistical analysis given that they presented with recurrence of retinal detachment in the postoperative period and were re-operated. A total of 70 eyes from 70 patients satisfied the study criteria and were included in the analysis.

The clinical demographics and the surgical characteristics are summarized in Table 1. There were 43 males and 27 females with a mean age ± standard deviation (SD) of 65.57 ± 10.89 years (range = 43-88 years old). In summary, 34 right and 36 left eyes were included in the study and the average number of retinal tears detected intra-operatively was 3.55 ± 3.61, ranging between 1 and 18 tears. Gas was utilized as intraocular tamponade agent in 64 cases (C3F8 in 58 and SF6 in six cases), while silicone oil was used in the remaining six cases. The mean duration of patients’ postoperative posturing was 7.27 ± 2.6 days. No significant differences were encountered in the patients' demographics and the clinical factors among the three groups.

**Table 1:** Patients' demographics and clinical characteristics.

Eyes (RE/LE)	70 (34/36)
Age (+/- SD)	65.57 (+/- 10.89)
Males/Females	43/27
No of retinal tears (+/- SD)	3.55 (+/- 3.61)
Treatment Platform	Group A: 28 eyes
	Group B: 22 eyes
	Group C: 20 eyes
Retinal tears treatment	Cryotherapy: 52
	Cryotherapy + Laser: 18
Residual SRF (yes/no)	22/48
Tamponade	C3F8 gas: 58 eyes
	SF6 gas: 6 eyes
	Silicone oil: 6 eyes
Duration of post-op face-down positioning	<= 7 days: 52 eyes
	> 7 days: 18 eyes

**Figure 1:**
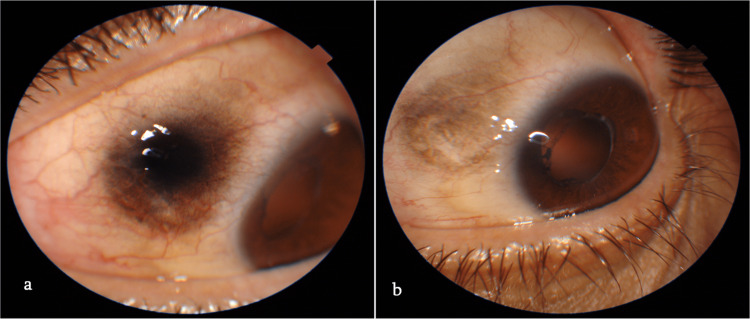
Postoperative conjunctival pigmentation one month (a) and six months (b) after PPV using 25G non-valved cannulas. PPV, pars plana vitrectomy

Forty-five out of 70 eyes (64.3%) demonstrated conjunctival pigmentation after vitrectomy at least in one time-point of the study (Figure 1). Age, gender, and duration of post-operative face-down positioning were not significantly correlated with the presence of conjunctival pigmentation in any of the treatment groups (p > 0.05). The clinical factors affecting the occurrence (part I model) and the surface area (part II model) of conjunctival pigmentation in photo measurements at each time point are summarized in Tables 2-3, respectively. Patients in the group B or group C were less likely to have conjunctival pigmentation [OR 0.26 (95% CI 0.03; 1.34), 0 (0; 0.04) respectively] at all follow-up visits within the first six months after the surgery, compared to patients from the group A. Two eyes from the group C presented with positive pigmentation photo measurement.

**Table 2:** Logistic regression analysis for the association between the risk factors and the presence of conjunctival pigmentation.

Logistic regression analysis			
1 month			
Parameter		OR (95% CI)	p value
Treatment group	Group A	12.5 (3.73; 77.69)	<0.001
	Group B	0.26 (0.03; 1.34)	0.013
	Group C	0 (0; 0.04)	<0.001
3 months			
Parameter		OR (95% CI)	p value
Treatment group	Group A	11.8 (3.7; 59.46)	<0.001
	Group B	0.44 (0.07; 2.36)	0.33
	Group C	0.01 (0; 0.05)	<0.001
Tamponade	SF6	0.09 (0.01; 0.67)	0.02
	Silicone	4.83 (0.26; 142.58)	0.29
6 months			
Parameter		OR (95% CI)	p value
Treatment group	Group A	5.75 (2.21; 19.61)	0.001
	Group B	0.37 (0.08; 1.45)	0.16
	Group C	0.02 (0; 0.1)	<0.001
1 year			
Parameter		OR (95% CI)	p value
Residual SRF	No	0.68 (0.37; 1.21)	0.19
	Yes	5.89 (1.84; 23.12)	0.005

**Table 3:** Linear regression analysis for the association between the risk factors and the area of conjunctival pigmentation.

Linear regression analysis				
1 month				
Parameter			Estimate (95% CI)	p value
Intercept			1.93 (-0.92; 4.79)	0.18
Number of RTs			0.92 (0.37; 1.47)	0.001
3 months				
Parameter			Estimate (95% CI)	p value
Intercept			3.63 (0.88; 6.37)	0.01
Number of RTs			0.79 (0.35; 1.24)	<0.001
Treatment Group	Group B		-4.86 (-8.22; -1.5)	0.005
	Group C		-6.29 (-14.14; 1.57)	0.113
6 months				
Parameter			Estimate (95% CI)	p value
Intercept			3 (0.59; 5.41)	0.01
Number of RT			0.61 (0.24; 0.99)	0.002
Treatment Group	Group B		-3.94 (-6.87; -1.01)	0.009
	Group C		-5.05 (-11.57; 1.46)	0.12
1 year				
Parameter			Estimate (95% CI)	p value
Intercept			2.19 (0.23; 4.15)	0.03
Number of RT			0.35 (0.06; 0.64)	0.02
Treatment Group		Group B	-2.6 (-4.86; -0.33)	0.02
		Group C	NA	
2 years				
Parameter			Estimate (95% CI)	p value
Intercept			-1.53 (-11.03; 7.97)	0.68
Number of RT			0.81 (-0.46; 2.08)	0.15

The SF6 gas was negatively correlated with positive photo measurement at 3 months [OR 0.09 (95% CI 0.01; 0.67)], whereas the presence of residual SRF was a risk factor for pigmentation one year postoperatively [OR 5.89 (95% CI 1.84; 23.12)]. Notably, nearly all cases that presented with residual subretinal fluid after vitrectomy (21/22, 95.5%) demonstrated presence of conjunctival pigmentation 3 months postoperatively (p < 0.001, Fisher’s exact test). The analysis did not reveal any statistically significant predictors for the two-year interval. The linear regression analysis revealed that the number of retinal tears was significantly associated with the surface pigmented area on the conjunctiva upon all visits during the 2 years of follow up.

No serious postoperative complication was observed in any of the 70 eyes included in the study. No incidence of sclerotomy leakage or IOP less than 6 mmHg was recorded during the 2 years follow-up period. Moreover, no cases of postoperative endophthalmitis or inflammation were noted.

## Discussion

To the authors’ knowledge, there are few cases in the literature reporting conjunctival pigmentation at the sclerotomy site following vitrectomy and only one study presents the possible contributing factors on patients operated for various reasons. Our study is the first to investigate the risk factors for conjunctival pigmentation following vitrectomy for retinal detachment comparing three different surgical techniques/platforms with the use of valved or non-valved cannulas. The main advantages of valved cannulas are maintenance of IOP and intraocular fluidic stability during vitrectomy, with equal surgical success and complication rates, but improved anatomical outcomes compared to non-valved surgery [18-19].

The benign origin of these conjunctival lesion has been confirmed by various reports in the literature. Smiddy et al. published a case of conjunctival and episcleral dark-brown pigmentation around the sclerotomy site following 20G PPV [16]. The histological analysis of the lesion showed the presence of melanin granules in the extracellular space and in macrophages. Based on the location it is clear that the pigmented area is not a conjunctival lesion but that melanin originates from the ruptured internal part of the eye and possibly the ruptured uveal cells. Park et al. also presented three cases of successfully treated rhegmatogenous retinal detachments with conjunctival pigmentation 4-5 days postoperatively at the sclerotomy site of 25G Torpedo Mini-Light chandelier illumination system [14]. They conducted light microscopic examination that confirmed the abundance of melanin pigments in the substantia propria of macrophages, whereas the presence of nevus cells, melanocytes or melanoma cells were excluded. Similar histologic findings were also presented in a case with bilateral conjunctival dense pigmentation after sutureless vitrectomy for vitreous hemorrhage at all three sclerotomy sites in both eyes a month postoperatively, confirming the benign nature of lesions and the intraocular origins of pigmentation [15]. The findings of our study have not been confirmed by histological analysis but the location of increased pigmentation at the post-vitrectomy period as well as the exclusion of previous conjunctival lesion contributes to the diagnosis of postoperative vitrectomy-related pigmentation.

Another finding supportive of the benign nature of pigmentation demonstrated in our study was the clear reduction in surface area of conjunctival pigmentation during the follow-up period, whereas complete absorption of pigment was also revealed in the majority of eyes. More specifically only six patients presented with positive photo measurement at the 2 years postoperative visit, concluding that this is probably a temporary cosmetic complication after vitrectomies and no treatment is required.

The significance of conjunctival pigmentation as a prognostic sign of sympathetic ophthalmia in sensitized patients has also been mentioned by Cha et al. [20]. The authors reported a case with dense conjunctival pigmentation around the sclerotomy sites following sutureless 23G vitrectomy performed for rhegmatogenous retinal detachment. The diagnosis of vitrectomy-related sympathetic ophthalmia was made and they hypothesized that conjunctival pigmentation may be driven by the exposure of uveal antigens to patient's immune system. The finding of our study cannot confirm this hypothesis, given that there was not any incidence of sympathetic ophthalmia in any of 70 patients during the two follow-up years.

Leakage through the sclerotomies would be a reasonable explanation for conjunctival pigmentation. The role of the oblique incisions on sclerotomy leakage has been implicated in various studies. Singh et al. studied the leakage of straight and angled incisions following 23G and 25G PPV [21]. They found no significant difference between 23G and 25G incisions. However, angled incisions could be desirable in terms of conjunctival pigmentation at the sclerotomy sites, given that 10.8% of straight incisions and 5.7% of angled wounds leaked after the surgery. In all our cases, oblique trocars insertion was applied without any signs of wound leakage in the postoperative period. In contrast to these findings, Bourla et al. showed that more than one-third of retinal detachment cases treated with gas tamponade demonstrated leakage despite the oblique 25G trocars direction of sclerotomies [22].

The role of vitreous base dissection is another factor that has also been described in many studies. Woo et al. reported the plugging role of vitreous remnants during sutureless vitrectomy at the sclerotomy site and highlighted the meticulous vitreous base dissection as one of the risk factors responsible for sclerotomy leakage following 23G vitrectomy [7]. In our study, the nature of retinal detachment cases required meticulous vitreous base shaving that was performed by the same surgeon in all cases from the three groups. Nagpal et al. have suggested the hypothesis that the vitreous around the 20-G sclerotomy is reachable and can be easily removed with the cutter [23]. In contrast, in case of sutureless vitrectomy systems, the trocars extend into the vitreous cavity and the surrounding vitreous cannot be completely cleaned. Upon removal of trocars, the intravitreal fluid clogs the port from inside and impede further leakage to the subconjunctival space. In contrast, the study from Abulon and Charles conducted in post-mortem rabbits demonstrated that the valved cannulas contribute to the intraoperative IOP stability as well as the significant lower rate of vitreous incarceration compared to the non-valved ones [18].

In the present study the number of retinal tears was a risk factor to the surface area of conjunctival pigmentation. Given that the release of pigment in the vitreous cavity is closely associated with retinal tear formation, the increased number of tears could therefore contribute to higher rate and area of pigmentation [24]. This seems a reasonable explanation despite not yet investigated by others. We also concluded that the presence of SRF at the end of the vitrectomy is possibly associated with increased pigmentation. This association has not been previous mentioned in the literature. We suppose that the raised concentration of pigment in the residual fluid could potentially pass, with the aid of surface tension from the tamponade, through the sclerotomy in the first postoperative days and cause positive pigmentation sign to the patients' conjunctiva.

The role of C3F8 tamponade in conjunctival pigmentation has been demonstrated by Park et al., who showed that the conjunctival pigmentation coming from sclerotomy sites was positively related to the C3F8 tamponade following 23G microincisional vitrectomy surgery (MIVS) for various indications [25]. In addition, the mean maximum pigmented area of eyes treated with C3F8 tamponade was greater than those of eyes treated without C3F8 tamponade. Our study demonstrates the possible protective role of the SF6 tamponade to the postoperative appearance of pigmentation. However, the small number of cases operated with SF6 as well as the choice to use it in less complicated cases with only one superior break could explain this finding.

The present study has some limitations regarding the interpretation of our results. Several studies have demonstrated that cryotherapy retinopexy promotes the dispersion of viable RPE cells in addition to extracellular melanin around the margins of the applied area, which could prolapse through the sclerotomy sites [26-27]. However, our study could not define the role of retinopexy on conjunctival pigmentation, given that all cases have been treated with cryotherapy or combination of cryotherapy/endolaser. Moreover, some other factors that could also contribute to the presence of pigmentation following vitrectomy, such as the duration of vitrectomy and the maximum of intraoperative IOP, have not been assessed in the present study. The limited number of patients included in this study makes the determination of all possible risk factors and their accurate correlation with conjunctival pigmentation difficult.

## Conclusions

In conclusion, the evolution of surgical instruments and techniques in vitrectomy has played an important role in the reduction of many postoperative complications, including postoperative conjunctival pigmentation also. The most significant predisposing factors for this complication in patients treated for rhegmatogenous retinal detachment are the non-valved trocars, the presence of residual SRF at the end of the surgery, and the number of retinal tears. All surgeons should be, therefore, aware of this cosmetic complication that contributes not only to the differential diagnosis of malignant lesions but is also important for the right preoperative consultation of high-risk patients.
